# Incidence and Temporal Variations of Bone and Soft Tissue Cancers in the Golestan Province, Northern Iran, 2004–2016

**DOI:** 10.34172/aim.2023.11

**Published:** 2023-02-01

**Authors:** Aida Soghi, Mehdi Aarabi, Seyyed Mehdi Sedaghat, Faezeh Salamat, Fatemeh Ghasemi-Kebria, Gholamreza Roshandel, Nafiseh Abdolahi

**Affiliations:** ^1^Golestan Rheumatology Research Center, Golestan University of Medical Sciences, Gorgan, Iran; ^2^Deputy of Public Health, Golestan University of Medical Sciences, Gorgan, Iran; ^3^Golestan Research Center of Gastroenterology and Hepatology, Golestan University of Medical Sciences, Gorgan, Iran

**Keywords:** Bone cancer, Golestan, Incidence, Iran, Soft tissue cancer

## Abstract

**Background:**

This study was conducted to evaluate the epidemiological features of bone and soft cancers in the Golestan province, Northern Iran from 2004 to 2016.

**Methods:**

This is a descriptive cross-sectional study. All patients with primary bone and soft tissue cancers between 2004 and 2016 were included. Data were obtained from Golestan population-based cancer registry (GPCR). We calculated age-standardized incidence rates (ASRs) and reported the rates per 100000 person-year. Estimated annual percent change (EAPC) was also calculated to assess temporal trends in incidence rates of these cancers.

**Results:**

The ASRs of bone cancers and soft tissue cancers were 1.33 and 1.43 per 100000 person-year, respectively. This study also showed that the ASR of bone cancer was higher in men (1.51) than women (1.15). The ASR of soft tissue cancers in the urban population (1.58) was higher than rural (1.27), and was lower in women (1.37) than men (1.49). Two peaks were seen in the incidence of bone cancer. The first peak was in the age group of 10 to 20 years and the second was in patients over 60. We did not find significant temporal trends in the incidence of bone (EAPC=-1.14; *P*>0.05) and soft tissue cancers (EAPC=-2.73; *P*>0.05) during the study period.

**Conclusion:**

Epidemiological features of bone and soft tissue cancers including gender, age and place of residence should be considered by health policy makers in designing cancer control programs.

## Introduction

 In 2020, approximately 19.3 million patients were diagnosed with cancer and approximately 10 million deaths from cancer were reported in the world. The three most commonly reported cancers were breast, lung and colorectal cancers, in decreasing order.^1^

 The total incidence of cancers in Iran is 110 and 98 per 100 000 in men and women, respectively, and the mortality rate in men and women is 65 and 41.1 per 100 000, respectively. In Iran, the most common cancers are gastric, prostate, bladder, colorectal and esophageal cancers among men, and breast, colorectal, gastric, esophageal, and thyroid cancers among women.^2^

 In general, primary bone tumors are rare (include less than 1% of detected tumors in a year) but have relatively high mortality and morbidity. The most common bone tumors are osteosarcoma, chondrosarcoma and Ewing sarcoma, respectively. Osteosarcoma and Ewing sarcoma together account for approximately 50% of malignant bone tumors in children and young people, which have major effects on the lives of patients and their families. In addition to the burden of the disease, the financial burden of these diseases has also special importance. Besides the growing incidence of these cancers, many of the costs of these diseases are incalculable, so the epidemiological study in this field is undeniably justified.^3^

 Early detection of bone cancers is a challenging issue because of delayed diagnosis and non-specified symptoms (for example, common musculoskeletal pain), which can lead to late diagnosis.^4^

 Over the past few decades, the number of these cancers has increased dramatically. Part of this is related to aging and population growth, which requires cancer growth control programs. The basis for addressing this problem is having access to epidemiological information from cancer registry databases. The first population-based cancer registration began in Europe in the 1920s and a few years later in the United States.^5^

 Soft tissue cancers have a wide range of pathologies (close to 70 types) and can affect people of any age and in any anatomical location.^6^ In Europe, the prevalence of soft tissue cancer is higher than the prevalence of bone cancer, and both types are more common in men than women.^7^

 Most types of bone cancers occur sporadically.^8^ In a study conducted on dogs, they concluded that stress due to being overweight, borne on bones during the age of growth, can increase the risk of primary bone cancer, which was also generalized to humans.^9^

 Radiation can be a risk factor for both bone and soft tissue cancer. Also, the risk of soft tissue cancer in immunocompromised people (acquired or congenital), is higher than others with a normal immune system.^10^

 The share of bone cancer cases in mortality is disproportionately high among adolescents and young people in the age of 15 to 24 years. In developed countries, including the United States and the United Kingdom, primary bone tumors are the third common cause of death from cancer among people of young age after leukemia and central nervous system cancer.^11^

 Due to the low prevalence and incidence of soft tissue sarcoma (STS), in many studies, it may even be ignored. The existing studies have been performed in developed countries.^12^ There is no accurate estimate of the prevalence and incidence of primary bone cancer in developing countries. Health system managers and planners in different countries need to be aware of the prevalence and incidence of these diseases to control the mortality and morbidity rates and costs.^13^

 Many studies have reported that primary bone cancer is age-related and has a higher prevalence in younger age based on the results of many studies; this has led to the analysis of the incidence of bone cancers in different age groups to investigate the effect of age.^14^

 Epidemiologically, lack of a single method for reporting sarcomas has led to significant variation in reported incidence rates and temporal changes.^15^ Previously, extensive studies on gastrointestinal cancers (which are among the most common cancers in the region) have been conducted in the Golestan province in terms of epidemiology, temporal changes, etc. So far, no report on the epidemiology and incidence and temporal changes of bone and soft tissue cancers in this province has been published and the present study was conducted with this purpose during 2004-2016 in the Golestan province.

## Materials and Methods

 The present study is a descriptive cross-sectional study. The study population includes residents of the Golestan province. The Golestan province is located in northeastern Iran, with a land area of 20,438 km^2^, which is about 1.3% of the total land area of Iran. Almost 50% of its population live in urban areas. All patients with primary bone and soft tissue cancers who developed these cancers between 2004 and 2016 participated in the study after filling out the informed consent form. After receiving the patients’ information from the Golestan population-based cancer registry (GPCR), the necessary preparations were made for data analysis. For this purpose, the population information of the province in the studied years was obtained from the Statistics and Information unit of the Golestan University of Medical Sciences and then the crude incidence of cancers was calculated. In the next step, using the world standard population with a direct standardization method,^16,17^ the age-standardized rates of cancers were calculated and reported separately for the study variables, and then tables and graphs were used to describe and report the findings.

 SPSS version 16 was used for data analysis and tables and graphs were used to describe the data. Age-specific rates, crude rates and age-standardized incidence rates (ASRs) and 95% confidence intervals (CI) of ASRs were calculated and reported per 100 000 person-year. Estimated annual percent changes (EAPCs) and their 95% CIs were calculated to assess the temporal trends in the incidence rates of cancer during the study period.

## Results

 In this study, 569 cases of bone and soft tissue cancers were registered, of which 274 cases were bone cancers and 295 cases were soft tissue cancers. The mean age of patients with bone cancer was 39.46 and the standard deviation was 21.6 years; the mean age of patients with soft tissue cancer was 40.87 and the standard deviation was 19.9 years.

 The distribution of diagnostic methods in bone and soft tissue cancer in the Golestan province, during 2004–2016 is shown in Table 1. The results show a high proportion of pathology confirmed cases and a low proportion of death certificate only (DCO), suggesting high data quality.

**Table 1:** Distribution of Diagnostic Methods in Bone and Soft Tissue Cancers in the Golestan Province, 2004–2016

		**Bone Cancer, ** **No. (%)**	**Soft Tissue Cancer, ** **No. (%)**
Diagnostic methods	Pathology	188 (68.6)	256 (86.8)
	Clinical/paraclinical	67 (24.5)	38 (12.9)
	Death certificate only	19 (6.9)	1 (0.3)

 The ASRs (95% CI) of bone cancers and soft tissue cancers were 1.33 (1.17–1.49) and 1.43 (1.25–1.61) per 100,000 person-year, respectively. Table 2 shows the number, percent, crude rates, ASR and 95% CI of the ASR (per 100 000 person-year) of bone and soft tissue cancer in the Golestan province during 2004-2016. Our results suggested higher rates of bone and soft tissue cancers in males as well as higher rates of soft tissue cancers in urban areas.

**Table 2:** Number, Percent, Crude Rate, Age-Standardized Incidence Rate and 95% Confidence Interval of Bone and Soft Tissue Cancer in the Golestan Province, Iran, 2004-2016

		**Variable**	**Number**	**Percent**	Crude Rate^*^	ASR^*^	95% CI of ASR^*^	
							**Lower**	**Upper**
Bone cancer	Gender	Male	153	55.8	1.36	1.51	1.26	1.76
		Female	121	44.2	1.08	1.15	0.93	1.37
	Place of residence	Urban	141	51.5	1.23	1.34	1.1	1.58
		Rural	133	48.5	1.21	1.36	1.12	1.6
Soft tissue cancer	Sex	Male	148	50.2	1.32	1.49	1.24	1.74
		Female	147	49.8	1.31	1.37	1.13	1.61
	Place of residence	Urban	162	54.9	1.42	1.58	1.33	1.83
		Rural	133	48.1	1.20	1.27	1.05	1.49

ASR, age-standardized incidence. * Per 100 000 person-year.

 The number, crude rate and ASR of bone and soft tissue cancers by the year of diagnosis are presented in Tables 3 and 4, respectively.

**Table 3:** Number, Crude Rate, Age-Standardized Incidence Rate and 95% Confidence Interval for ASR of Bone Cancers in the Golestan Province, Iran, 2004–2016.

**Year**	**Number**	Crude rate*	ASR*	95% CI of ASR^*^	
				**Upper**	**Lower**
2004	22	1.42	1.53	2.26	0.80
2005	26	1.64	1.95	2.77	1.13
2006	19	1.17	1.21	1.80	0.62
2007	18	1.09	1.15	1.72	0.58
2008	26	1.55	1.59	2.24	0.94
2009	16	0.93	1.27	1.92	0.62
2010	13	0.75	0.79	1.24	0.34
2011	21	1.18	1.26	1.83	0.69
2012	18	1.00	1.00	1.49	0.51
2013	22	1.21	1.47	2.10	0.84
2014	26	1.42	1.62	2.25	0.99
2015	26	1.41	1.50	2.09	0.91
2016	21	1.12	1.20	1.73	0.67

ASR, age-standardized incidence. * Per 100 000 person-year.

**Table 4:** Number, Crude Rate, Age-standardized Incidence Rate and 95% Confidence Interval for ASR of Soft Tissue Cancers in the Golestan Province, Iran, 2004–2016.

**Year**	**Number**	Crude Rate^*^	ASR^*^	95% CI of ASR^*^	
				**Upper**	**Lower**
2004	14	0.90	1.14	1.77	0.51
2005	20	1.26	1.33	1.94	0.72
2006	30	1.86	2.35	3.25	1.45
2007	30	1.82	2.34	3.24	1.44
2008	30	1.79	1.67	2.3	1.04
2009	22	1.29	1.47	2.12	0.82
2010	16	0.92	1.07	1.62	0.52
2011	21	1.18	1.20	1.75	0.65
2012	19	1.06	1.17	1.72	0.62
2013	20	1.10	1.26	1.83	0.69
2014	18	0.98	1.03	1.52	0.54
2015	24	1.30	1.31	1.86	0.76
2016	31	1.66	1.57	2.16	0.98

ASR, age-standardized incidence. * Per 100 000 person-year.

 The EAPC (95% CI) was -1.14 (-12.77–12.04) (*P* value > 0.05) for bone cancer and -2.73 (-13.80–9.77) (*P* value > 0.05) for soft tissue cancer. The findings suggested that there were no significant temporal trends in the incidence rates of bone and soft tissue cancers in the Golestan province between 2004 and 2016.

**Figure 1:**
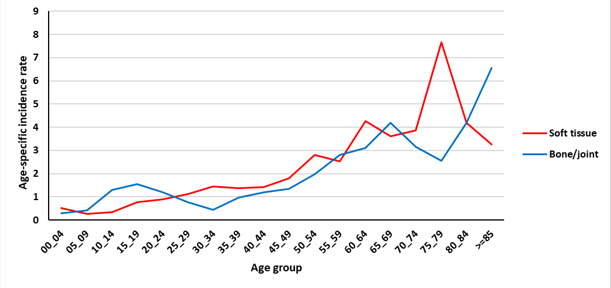
The Age-Specific Incidence of Bone and Soft Tissue Cancer in Golestan Province During the Years 2004 to 2016 in Different Age Groups.

Figure 1 shows the age-specific incidence rate (per 100 000 person-year) of bone and soft tissue cancers in the Golestan province during 2004 - 2016. Our results suggested two peaks for the age specific rates of bone cancers. The first peak is observed around the age of 20 and the second peak is observed after the age of 60 years.

**Figure 2:**
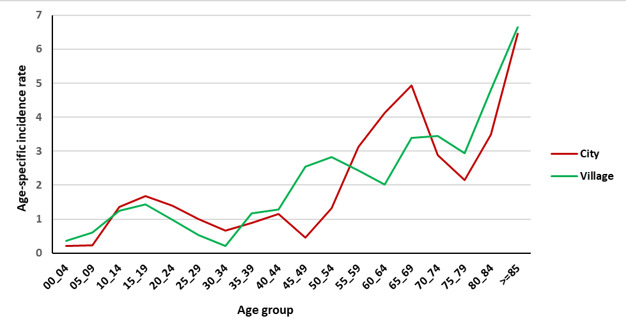
The Age-Specific Incidence of Bone and Joint Cancer in Golestan Province During the Years 2004 to 2016 (City and Village).

**Figure 3:**
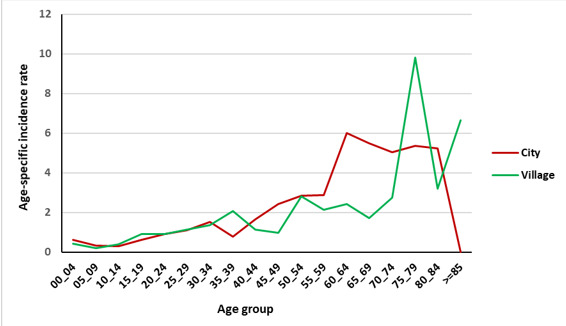
The Age-Specific Incidence of Soft Tissue Cancer in Golestan Province During the Years 2004 to 2016 (City and Village).

**Figure 4:**
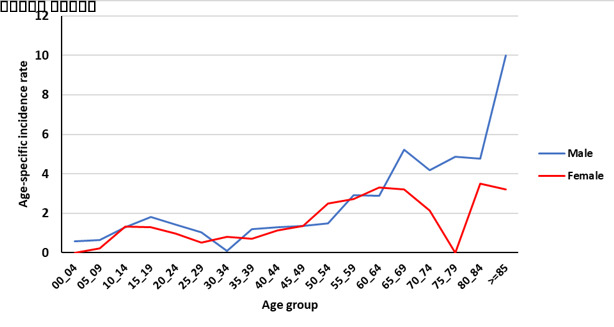
The Age-Specific Incidence of Bone and Joint Cancer in Golestan Province During the Years 2004 to 2016 by Gender.

**Figure 5:**
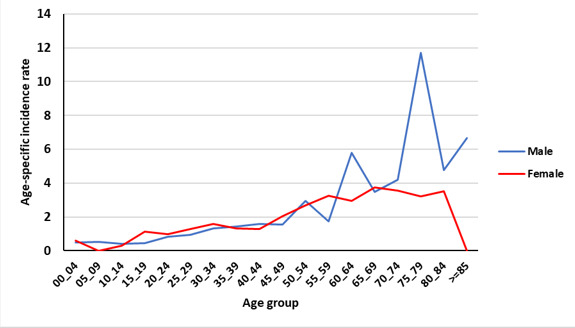
The Age-Specific Incidence of Soft Tissue Cancer in Golestan Province During the Years 2004 to 2016 by Gender.

Figures 2 and 3 show the age-specific incidence rate (per 100 000 person-year) of bone and soft tissue cancer in the Golestan province during 2004–2016 by residence area. Finally, in Figures 4 and 5, the age-specific incidence rate (per 100 000 person-year) of bone and soft tissue cancer in the Golestan province during 2004-2016 are shown by sex.

## Discussion

 The present study is a descriptive cross-sectional study that was performed on 569 patients with bone and soft tissue cancer in the Golestan province during the years 2004 to 2016. In this study, various parameters such as age-specific incidence rate and incidence rate by age, sex, place of residence (city or village) etc have been reported.

 In the present study, the ASRs of bone cancer in men and women were reported at 1.51 and 1.15 per 100 000 person-year, respectively. According to the International Agency for Research on Cancer, the ASRs of bone cancer in men and women were respectively 1.1 and 0.8 in the United States, 1.9 and 1.3 in China, 0.4 and 0.5 in India, 1.1 and 2 in Japan, 0.8 and 1.2 in Qatar, 1 and 0.9 in Turkey, 1.1 and 0.9 in Germany, 1.3 and 0.9 in the UK, 0.9 and 0.9 in Bahrain and 1.1 and 0.7 in Australia.^18^

 From the epidemiological point of view, lack of a single method for reporting sarcomas has led to significant variation in incidence rates and they are sometimes misdiagnosed as carcinomas of the involved organ. As a result, 30% of sarcomas have an inappropriate classification in the initial diagnosis, which is one of the problems in the epidemiological study of this type of cancer.^15^ This may be a reason for the difference in the incidence rates across countries with similar levels of health system.

 In the present study, the most common type of bone cancer was osteosarcoma with a proportion of 23.7% while in the study conducted in Isfahan, a proportion of 15.1% was reported.

 In the present study, as in other studies, the age-specific incidence of primary bone tumors had two peaks. The first peak is observed around the age of 20 and then after a relative decrease, the second peak is observed again at the age of over 60 years. In other similar studies, including the Isfahan study, two age peaks were observed, one in the age group of 10 to 20 years and the other in 40 to 50 years.^13^

 Similarly, in a study conducted in the United States from 1976 to 2005, the highest incidence was in the second decade of life with another peak observed in the age of 75 to 79, and overall, it occurred in women at younger ages. Although the osteosarcoma and chondrosarcoma incidence rate was higher in men over the years, the present study showed that in the last years of data collection, the incidence of bone cancer in women has increased. The increase in the incidence of chondrosarcoma in women plays a major role in raising the overall incidence and osteosarcoma plays a minor role in increasing the overall incidence in women.^19^

 In a study by Kumar and Gupta, data was collected from developed countries, and as in other studies, two age peaks for primary bone cancer were found. The first peak in the second decade of life was around 17 and 18 years of age and the second peak was from the age of 40 to 80 years and a steady increase was observed. The rate of age-specific incidence in the first decade of life between men and women was almost equal, but generally higher in men than women (by approximately 1.3 times). In the Golestan study, the incidence of bone cancer in the first peak in women and men was almost equal, but in general, men had a higher peak than women.^11^

 Changes in the standardized age incidence of bone cancer during the years 2004 to 2016 in the Golestan province did not have a specific ascending or descending pattern and remained almost constant during these years. A study was conducted in Taiwan from 2003 to 2010 on the incidence of primary bone cancers. The course of changes in the incidence in these years generally had an upward trend in men and women, which was higher in women than men in the study.^14^

 The ASRs of soft tissue cancer in men and women in the Golestan province were 1.49 and 1.37 per 100 000 person-year, respectively. The International Agency for Research on Cancer estimates the incidence of this cancer in men and women at 2.9 and 2.1 in the United States, 0.7 and 0.9 in China, 0.5 and 1.6 in India, 1.2 and 0.9 in Japan, 2.8 and 0.7 in Qatar, 2 and 1.8 in Turkey, 2.6 and 1.9 in Germany, 2.3 and 1.7 in the UK, 0.9 and 0.9 in Bahrain, and 2.6 and 1.8 in Australia.^18^

 In the Golestan study, the highest incidence was in the age of 75 to 79 years, and in another study conducted in the East, the peak was above the age of 85 years; both studies indicate an increase in the incidence at older ages.^20^

 Other studies conducted in the East include a study from China, which reviewed 39 900 STS cases in 2014. Of these, 5700 were related to GIST cases, which are also the most common cases. In this study, the overall incidence of STS and non-GIST cases in women was higher than men in both urban and rural areas. The lowest incidence is similarly in the ages of 5 to 9 years and its peak is reported at the age of 75 to 79 years; both age groups are consistent with the data of the Golestan study.^12^

 Changes in the age-standardized incidence of soft tissue cancer during different years in the Golestan province are as follows: it peaked during 2006 and 2007 and then decreased, and again in the final years of the study, in 1994 and 1995, began to increase. In another similar study, which was conducted on 5333 patients in Austria from 1984 to 2004, the incidence of changes during different years did not show a specific ascending or descending pattern.^21^

 In another study, 3,843 cases of soft tissue cancers in Taiwan from 2003 to 2011 were studied and annual specific incidence rate was calculated separately for men and women. The change pattern in women in the early years of research showed a slow decline and then a slow increase, and the changes were not significant over the years, while in men, these changes in general had an upward trend.^20^

 A large study was performed on 26,758 patients with soft tissue cancer between 1978 and 2001; between 1978 and 1983, and from 1996 to 2001, the ASR increased by 0.8 to 1.2% per year.^22^

 In a study conducted in France from 2000 to 2013 on soft tissue and bone sarcomas simultaneously, ASR levels increased in all types of sarcomas between 2000 and 2005, and thereafter from 2005, this rate had a constant level.^15^

 In a study which was conducted in Korea from 2007 to 2014, 7813 patients with STS were studied. The most common age group involved in this study were people between 50 and 59 years of age and more than half of the patients (52.1%) were female. The percentage of advanced types of cancers in the age group over 60 years was significantly higher than the younger ages. During this study, the annual incidence was calculated and an annual increase in incidence was observed. In fact, the overall incidence rate was 2.49 per 100 000 person - years, which was 5.64 in the age group over 70 years and higher than the younger age groups, and it was 1.07 in the age group of 18–29 years. These changes are to a large extent consistent with the results of the present study.^23^

 In Shanghai, China, a study was conducted on soft tissue and bone sarcomas simultaneously between 2002 and 2014, in which 87.9% were STS cases and 12.1% were bone sarcoma cases.

 In this study, most cases of sarcoma (60.9%) were reported in the age group of 20 to 64 years, 3.9% in the age group of 0 to 19 years and 35.2% in the age of 65 years.

 In general, the incidence of sarcomas increases with age; however, regarding bone sarcoma in this study, the highest incidence was reported at the age of 0 to 19 years.

 Regarding the incidence changes in this study, with an overview of sarcomas from 2002 to 2014, no significant changes in incidence were observed between men and women, but with further analysis, we noticed a decrease in the incidence of sarcomas such as leiomyosarcoma and fibrosarcoma, and the only observed increase in ASR pertained to women with STS. Therefore, in this regard, there is not much resemblance to the study in Golestan.^24^

 In conclusion, the present study is in fact the first study related to the epidemiology of bone and soft tissue cancers in the Golestan province. Paying attention to epidemiological findings can be useful in more effective planning to control these cancers in the province.

 It is suggested that health policy makers in the province pay special attention to the results of this study and other similar studies when planning to control these cancers. It is also suggested that additional studies should be designed and performed to determine the effective factors of this disease in the province and also to more accurately identify the other characteristics of these cancers, including the most common anatomical sites involved, etc.
